# How responsible leadership fosters ICU nurses’ innovation in China: a mediated model of perceived insider status and voice behavior

**DOI:** 10.3389/fpsyg.2026.1904747

**Published:** 2026-09-09

**Authors:** Ke Liu, Hua Du, Qianqian Cheng, Tingting Jiao, Yingying Tong, Yuewen Cai, Xinyue Zhang, Haitao Huang

**Affiliations:** 1 Neurological Intensive Care Unit, Department of Neurology, Kaifeng Central Hospital, Kaifeng, China; 2 Department of Nursing, West China School of Nursing, West China Hospital, Sichuan University, Chengdu, China

**Keywords:** innovative behavior, nurses, perceived insider status, responsible leadership, voice behavior

## Abstract

**Background:**

Responsible leadership may be associated with ICU nurses’ innovative behavior, but the underlying mechanisms remain unclear. This study examined the mediating roles of perceived insider status and voice behavior.

**Methods:**

A cross-sectional survey was conducted between January and February 2025 in three tertiary hospitals in northern China. A total of 372 ICU nurses were recruited using convenience sampling. Validated instruments were used to measure responsible leadership, perceived insider status, voice behavior, and innovative behavior. Structural equation modeling was employed to test the hypothesized model and mediating pathways.

**Results:**

The results indicated significant positive correlations among responsible leadership, perceived insider status, voice behavior, and innovative behavior (all *p* < 0.01). Responsible leadership not only directly influenced the innovative behavior of ICU nurses but also had indirect effects through the mediating roles of perceived insider status and voice behavior. Specifically, perceived insider status and voice behavior accounted for 22.56 and 21.05% of the total effect, respectively. In addition, perceived insider status and voice behavior played a sequential mediating role between responsible leadership and innovative behavior, with the chain mediation effect accounting for 8.77% of the total effect.

**Conclusion:**

This study highlights the critical role of responsible leadership in enhancing ICU nurses’ innovative behavior within the Chinese healthcare context. By fostering a strong sense of insider identity and encouraging upward communication, responsible leadership can create an inclusive, psychologically safe environment that empowers nurses to generate and implement innovative ideas. Hospital administrators should promote leadership practices that emphasize ethical responsibility, stakeholder engagement, and open communication to stimulate innovation and improve care outcomes in intensive care settings.

## 1 Introduction

Healthcare systems worldwide are placing increasing emphasis on innovation in clinical practice, particularly in high-stakes environments like intensive care units (ICUs) (Li-Ying et al., 2016). In China, rapid technological advances and shifting patient needs have created an urgent demand for innovative nursing practices to improve care quality and efficiency (Zhang et al., 2022). ICU nurses, who work under high clinical complexity and psychological pressure while managing critically ill patients, play a critical role in this transformation (Zhang et al., 2025). Their innovative behavior has been shown to enhance nursing quality and drive the sustainable development of healthcare (Li-Ying et al., 2016). Research indicates that when nurses introduce new ideas or methods, it not only improves patient outcomes and service quality but can also shorten hospital stays and reduce costs (Stewart et al., 2021; Saleh et al., 2025). Recognizing this, Chinese health authorities have highlighted innovation as a strategic priority in the medical sector. However, empowering nurses-especially those in ICUs—to consistently engage in innovative behavior remains a challenge (Li-Ying et al., 2016). There is growing consensus that leadership practices on the unit level are pivotal in fostering an environment where nurses feel encouraged to experiment and improve care delivery (Alluhaybi et al., 2024; Elbus et al., 2024).

Leadership is a key catalyst for staff performance and innovation in nursing organizations (Scully, 2015). Among various leadership styles, responsible leadership has gained attention for its ethical, inclusive, and stakeholder-oriented approach (Identity, 2007). It is defined as “the art and ability to establish, cultivate, and maintain trusting relationships with stakeholders both inside and outside the organization, as well as the responsibility to work together to achieve a meaningful and shared vision (Maak and Pless, 2006; Identity, 2007).” In the Chinese cultural context, where leaders are traditionally expected to embody moral integrity, serve as ethical role models, and skillfully balance competing stakeholder interests, responsible leadership resonates with local expectations of both virtue and pragmatism (Cheng et al., 2021). This style emphasizes not only achieving organizational goals but also harmonizing internal and external demands through accountability, coordination, and adaptive problem-solving (Han et al., 2019b; Cheng et al., 2021). Prior studies suggest that responsible leadership is associated with employees’ attitudes and behaviors (Han et al., 2019b). For example, evidence from a recent Chinese study showed that responsible leadership is positively associated with employees’ innovative behavior (Han and Ni, 2025). Such leaders may create supportive, fair, and value-driven work climates that motivate staff to go beyond routine tasks (Miska and Mendenhall, 2018). Yet, little research to date has examined whether responsible leadership is associated with innovative behavior among ICU nurses, particularly in China’s hospital environment. This gap merits attention given the pressing need for innovation in ICUs and the distinct cultural and organizational characteristics of Chinese healthcare settings (Zhao et al., 2023). Additionally, perceived insider status (PIS) reflects nurses’ sense of being valued and accepted as integral members of their organization, while voice behavior represents their proactive communication of suggestions aimed at improving clinical practice (Liu et al., 2021; Huang S. et al., 2025). Both are considered key interpersonal and behavioral resources that can facilitate innovation in teams (Guzman and Espejo, 2019; Yuan and Jiang, 2024). Although these constructs have been examined separately, their sequential roles remain unclear. Drawing on social identity theory, this study proposes an identity-to-action pathway in which responsible leadership is associated with innovative behavior through PIS and subsequent voice behavior. This framework clarifies how organizational belonging may be translated into proactive expression and innovation in the hierarchical and safety-critical ICU context. Therefore, this study examines the direct and serial mediating roles of PIS and voice behavior in the association between responsible leadership and innovative behavior among ICU nurses.

## 2 Background

### 2.1 Responsible leadership and innovative behavior

Responsible leadership, as defined by Voegtlin et al. refers to leaders balancing and coordinating multiple stakeholder interests through dialog and consultation to build mutually beneficial relationships inside and outside the organization (Voegtlin et al., 2012). Unlike traditional leadership theories that primarily focus on the leader–subordinate dyad (e.g., transformational leadership, ethical leadership, and servant leadership), responsible leadership emphasizes a multi-stakeholder perspective (Han et al., 2019a). Leaders are expected not only to pursue organizational objectives but also to address the expectations of diverse stakeholders, integrating economic, social, and ethical responsibilities into their decision-making processes (Han et al., 2019a; Haider et al., 2022). Empirical studies have demonstrated that responsible leadership can positively influence employee outcomes such as job performance, knowledge-sharing behavior, and environmentally sustainable practices (Lin et al., 2020; Haider et al., 2022; Tuan, 2022). In the Chinese cultural context, the concept of responsible leadership resonates strongly with traditional expectations that leaders should embody moral integrity, act as ethical role models, and skillfully balance competing interests (Cheng et al., 2021). Rooted in collectivist values, Chinese organizations often emphasize group harmony, respect for hierarchy, and relational interdependence, which may lead employees to avoid expressing divergent opinions to preserve interpersonal harmony (Ma et al., 2014). In this context, responsible leadership—which integrates moral authority with pragmatic coordination—can help bridge hierarchical gaps, create a psychologically safe environment, and encourage employees to participate in organizational improvement (Han and Ni, 2025). However, current research has predominantly focused on responsible leadership’s impact in business sectors (Mousa and Arslan, 2023), with limited evidence concerning its role in healthcare, particularly among ICU nursing staff. Given the complexity of ICU work and the urgent need for continuous innovation, understanding how responsible leadership can promote innovative behavior among ICU nurses is both theoretically and practically significant.

Based on these considerations, we propose *Hypothesis 1*: *Responsible leadership positively predicts ICU nurses’ innovative behavior.*

### 2.2 The potential mediating role of perceived insider status (PIS)

According to organizational identity theory, perceived insider status (PIS) reflects employees’ subjective sense of being accepted as valued members within their organization (Guo et al., 2022). When leaders demonstrate fairness, support, and respect, employees are more likely to feel integrated into the organizational community, thus enhancing their perceived insider status (Guo et al., 2022). Positive leadership behaviors create an inclusive environment that fosters belonging, recognition, and trust, encouraging employees to identify more closely with organizational goals (Wijewardena et al., 2014). Previous studies have shown that leadership styles such as ethical and inclusive leadership are positively associated with perceived insider status, as supportive leaders strengthen employees’ sense of being insiders (Zhong et al., 2019; Lee et al., 2022).

Additionally, Employees who perceive themselves as insiders are more willing to actively contribute to organizational development (Wang and Kim, 2013). In the Chinese cultural context, the concept of perceived insider status is closely linked to traditional notions of belonging and collective identity (Wang and Kim, 2013). When employees perceive themselves as organizational insiders, they tend to adopt a “host mentality” and assume ownership responsibilities within the organization, thus exhibiting more behaviors that benefit organizational goals (Zhang K. et al., 2024). Existing studies have shown that employees with high PIS are more likely to engage in proactive attitudes and extra-role behaviors, as inclusion fosters trust and commitment (Hui et al., 2015; Zhang K. et al., 2024). In high-pressure environments like the ICU, where hierarchical structures may suppress open expression, enhancing nurses’ sense of insider identity can help them overcome psychological barriers and promote active involvement in organizational improvement.

Based on the above, we propose *Hypothesis 2*: *Perceived insider status mediates the relationship between responsible leadership and ICU nurses’ innovative behavior.*

### 2.3 The potential mediating role of voice behavior

Voice behavior refers to employees’ voluntary expression of constructive suggestions, concerns, or opinions aimed at improving organizational functioning (Huang S. et al., 2025). As an important proactive behavior, voice reflects employees’ willingness to challenge existing practices and propose new ideas for organizational development (Huang S. et al., 2025). Leaders play a key role in shaping voice behavior, as their attitudes and actions determine whether employees feel psychologically safe to speak up (Chen and Hou, 2016). Studies have shown that leadership styles such as ethical and inclusive leadership can promote voice behavior by creating open, supportive communication environments where employees feel their input is valued (Chen and Hou, 2016; Qi and Liu, 2017).

In the context of responsible leadership, which emphasizes fairness, dialog, and stakeholder coordination, leaders are likely to encourage open expression and listen to diverse perspectives (Cheng et al., 2021). By balancing the needs of different stakeholders and fostering trust, responsible leaders can reduce subordinates’ fear of negative consequences from speaking up, thereby stimulating voice behavior (Uzum et al., 2025). Particularly in hierarchical and collectivist cultures such as China, where employees may hesitate to express dissenting views to maintain group harmony, responsible leadership can help break communication barriers and empower employees to share innovative suggestions (Yue et al., 2025). ICU nurses, who work in high-pressure environments with frequent procedural challenges, may particularly benefit from leadership that encourages their voice, as their frontline insights are essential for continuous clinical improvement. Therefore, this study hypothesizes that responsible leadership enhances ICU nurses’innovative behavior by promoting their voice behavior.

Based on this, we propose *Hypothesis 3*: *Voice behavior mediates the relationship between responsible leadership and ICU nurses’ innovative behavior*.

### 2.4 The relationship between PIS and voice behavior

PIS and voice behavior are both rooted in employees’ psychological perceptions of their organizational standing and their willingness to engage in proactive communication. PIS reflects the extent to which employees perceive themselves as valued, integral members of the organization, while voice behavior represents their readiness to express constructive suggestions for organizational improvement (Liu et al., 2021; Huang S. et al., 2025). Employees who perceive themselves as insiders are more likely to feel a sense of ownership and responsibility, motivating them to propose ideas that benefit the organization (Zhong et al., 2019). Existing studies indicate a significant positive relationship between PIS and voice behavior (Shalendra et al., 2021). Employees with higher insider status tend to experience stronger psychological safety and trust within their organization, which lowers their perceived risks of speaking up (Shalendra et al., 2021). Empirical evidence supports that when employees feel included and respected, they are more willing to express opinions and engage in voice behavior aimed at enhancing organizational processes (Qi and Liu, 2017). In China’s collectivist cultural environment, where maintaining group harmony often limits open expression, enhancing insider identity can help reduce hierarchical barriers and encourage constructive communication (Li and Sun, 2015). Therefore, this study hypothesizes that ICU nurses’ perceived insider status positively predicts their voice behavior.

**Figure 1:**
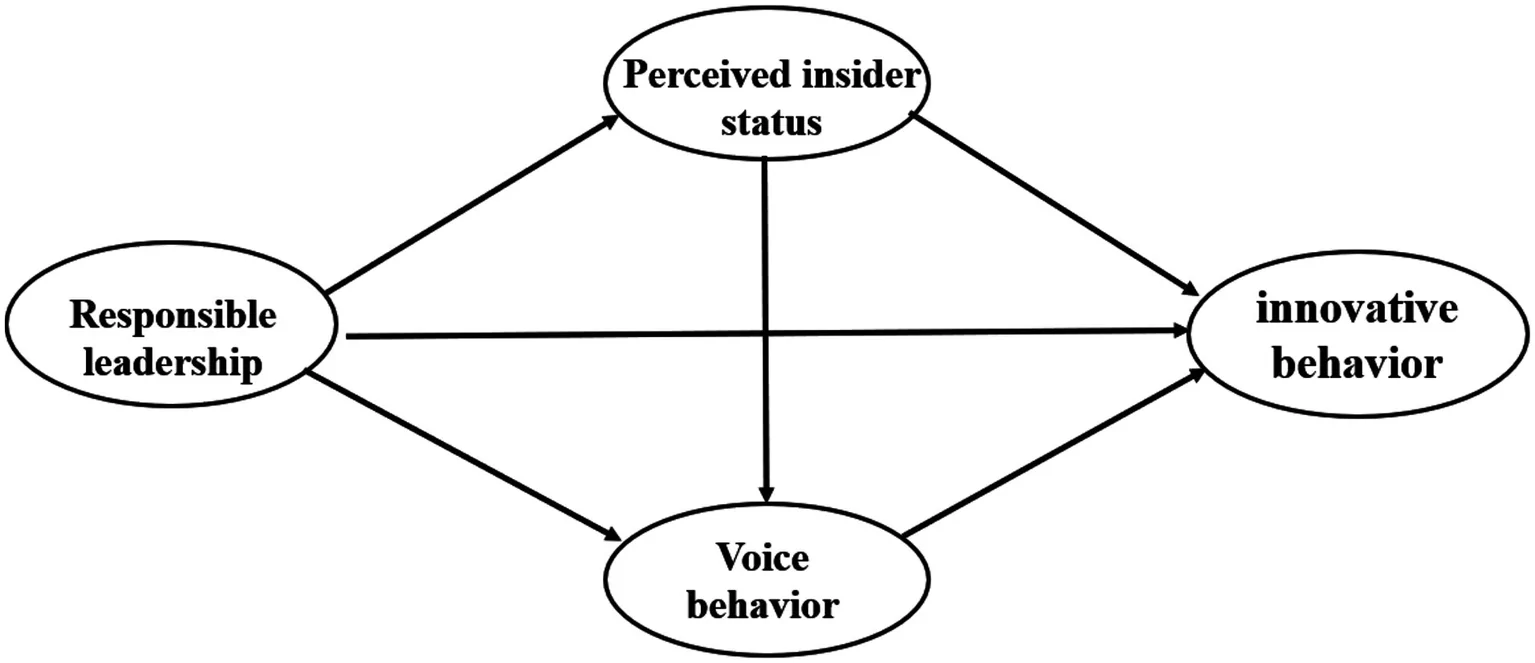
Conceptual framework of the hypothesized relationships.

Based on this, we propose *Hypothesis 4*: *Perceived insider status and voice behavior play a serial mediating role in the relationship between responsible leadership and ICU nurses’ innovative behavior.* The proposed conceptual framework is presented in Figure 1.

## 3 Methods

### 3.1 Design

This cross-sectional study was conducted following the STROBE (Strengthening the Reporting of Observational Studies in Epidemiology) guidelines (see Supplementary File S1).

### 3.2 Participants

This study adopted a convenience sampling method to recruit ICU nurses from three tertiary general hospitals in northern China. Each participating hospital typically operates multiple intensive care units, with nurse leaders overseeing approximately 30–50 ICU nurses responsible for direct patient care, professional development, and quality management activities. The inclusion criteria were as follows: (a) possession of a registered nurse qualification; (b) at least 1 year of independent clinical experience in an ICU; and (c) voluntary participation with informed consent. Nurses who were on extended leave or engaged in off-site training during the survey period were excluded.

The sample size was calculated using the formula *N* = 4U^2^αS^2^/δ^2^ (Ni et al., 2010), with α = 0.05, allowable error δ = 0.1, and pre-survey standard deviation S = 0.48. Accordingly, the required sample size was estimated as *N* = 4 × 1.96^2^ × 0.48^2^/0.1^2^ ≈ 354. To ensure adequate sample size accounting for potential invalid responses, 400 questionnaires were distributed, and 372 valid responses were ultimately collected after excluding incomplete or logically inconsistent questionnaires. The final sample size exceeded the recommended minimum of 200 cases required for structural equation modeling analysis, meeting the basic statistical requirements for model testing (Iacobucci, 2010; Kline, 2023).

### 3.3 Measurements

#### 3.3.1 Responsible leadership

The Responsible Leadership Scale, developed by Cheng et al. (2021). based on the Chinese cultural context, consists of four dimensions: self-discipline and consideration for others, social responsibility, interactive decision-making, and long-term strategic orientation. The scale includes 17 items, such as “My supervisor demonstrates altruism,” “My supervisor focuses on the long-term interests of the department,” and “My supervisor seeks consensus among affected stakeholders.” A five-point Likert scale is used, with scores ranging from 1 (strongly disagree) to 5 (strongly agree). Higher scores reflect a higher level of perceived responsible leadership among nurses. This scale has shown good reliability and validity in prior research (Han et al., 2024). In the present study, confirmatory factor analysis supported the four-factor structure of the scale (*χ*^2^/df = 2.45, CFI = 0.943, TLI = 0.931, RMSEA = 0.063, and SRMR = 0.041). Standardized factor loadings ranged from 0.68 to 0.89. The CR and AVE were 0.954 and 0.557, respectively. Cronbach’s α for the scale was 0.952.

#### 3.3.2 Perceived insider status

The Perceived Insider Status Scale, developed by Stamper and Masterson (2002), is a single-dimension measure comprising six items, including statements such as “I feel like I am an insider in my work organization,” “I feel a sense of belonging in my work organization,” and “I often feel left out at my work organization” (the latter representing one of three reverse-scored items). Responses are rated on a five-point Likert scale, ranging from 1 (strongly disagree) to 5 (strongly agree). Higher scores reflect a higher level of perceived insider status among nurses. The scale has demonstrated good reliability and validity in previous studies (Wang and Kim, 2013). In the present study, confirmatory factor analysis supported its unidimensional structure (*χ*^2^/df = 2.12, CFI = 0.987, TLI = 0.978, RMSEA = 0.055, and SRMR = 0.024). Standardized factor loadings ranged from 0.76 to 0.91. The CR and AVE were 0.938 and 0.716, respectively. Cronbach’s α for the scale was 0.936.

#### 3.3.3 Voice behavior

The Voice Behavior Scale, developed by Liang et al. (2012), consists of two dimensions: prohibitive voice and promotive voice. The scale includes 10 items, such as “I dare to point out problems at work even if it may affect colleague relationships,” “I speak up about issues that may cause work losses, even when others may disagree,” and “I proactively offer constructive suggestions to improve work and the organization.” A five-point Likert scale is used, with scores ranging from 1 (strongly disagree) to 5 (strongly agree). Higher total scores indicate a stronger willingness among nurses to engage in voice behavior. The scale has demonstrated good reliability and validity in previous studies (Yan and Xiao, 2016). In the present study, confirmatory factor analysis indicated that the two-factor model fitted the data adequately (*χ*^2^/df = 2.18, CFI = 0.956, TLI = 0.941, RMSEA = 0.057, and SRMR = 0.042). Standardized factor loadings ranged from 0.64 to 0.82. The CR and AVE were 0.873 and 0.516, respectively. Cronbach’s α for the scale was 0.857.

#### 3.3.4 Innovative behavior

This study adopted the Nurse Innovative Behavior Scale developed by Ling et al. (2012), which evaluates nurses’ engagement in innovative activities across three dimensions: idea generation, idea promotion, and idea realization. The scale comprises 10 items, such as “I am willing to generate solutions when encountering problems,” “I utilize resources to search for solutions,” and “I conduct research on new methods to obtain more information.” Each item is rated on a five-point Likert scale ranging from 1 (never) to 5 (very frequently), with higher scores indicating stronger willingness and frequency of engaging in innovative behaviors. The scale has demonstrated good reliability and validity in previous studies (Yan et al., 2020). In the present study, confirmatory factor analysis indicated that the three-factor model fitted the data adequately (χ^2^/df = 2.08, CFI = 0.961, TLI = 0.947, RMSEA = 0.054, and SRMR = 0.039). Standardized factor loadings ranged from 0.66 to 0.85. The CR and AVE were 0.887 and 0.526, respectively. Cronbach’s α for the scale was 0.868.

### 3.4 Ethical consideration

This study was approved by the Medical Ethics Committee of Kaifeng Central Hospital (approval no. 2025ks-kt004). The research strictly followed the ethical principles outlined in the Declaration of Helsinki. Prior to data collection, informed consent was obtained from the nursing management departments of the participating hospitals. All participants were fully informed about the purpose and significance of the study and were assured of the confidentiality and anonymity of their responses. Written informed consent was obtained from each participant, in accordance with the requirements of the institutional ethics review.

### 3.5 Data analysis

Data analysis was conducted using SPSS version 27.0 and AMOS version 25.0. Data were screened for missing values before analysis. Questionnaires containing missing responses were considered incomplete and excluded, and no data imputation was performed. Initially, descriptive statistics were computed to summarize participants’ demographic characteristics. The normality of continuous variables was assessed using the Kolmogorov–Smirnov test; a *p*-value > 0.05 indicated a normal distribution. For variables meeting normality assumptions, Pearson correlation analysis was applied to explore relationships among variables; otherwise, Spearman correlation was used. To examine the hypothesized mediation effects, structural equation modeling (SEM) was employed based on the covariance-based SEM approach, which is appropriate for hypothesis-driven path analysis. The variable “Perceived Insider Status” was processed using the item-to-construct balance method to optimize indicator-to-latent variable matching (Landis et al., 2000), while other variables retained their original measurement structure.

Model fit was evaluated using multiple indices: the chi-square to degrees of freedom ratio (χ^2^/df < 5), goodness-of-fit index (GFI > 0.90), adjusted goodness-of-fit index (AGFI > 0.90), comparative fit index (CFI > 0.90), incremental fit index (IFI > 0.90), root mean square error of approximation (RMSEA ≤ 0.08), and standardized root mean square residual (SRMR < 0.05) (Byrne, 2010; Kline, 2023). To test the significance of mediation effects, bias-corrected bootstrapping (5,000 iterations) was conducted. A 95% confidence interval (CI) not including 0 was interpreted as evidence of a significant indirect effect.

## 4 Results

### 4.1 Common method bias test

Harman’s single-factor test identified 11 factors with eigenvalues greater than 1. The first factor accounted for 31.57% of the total variance, which was below the recommended threshold of 40% (Podsakoff et al., 2003). These results suggest that common method bias was unlikely to substantially affect the findings of this study.

### 4.2 Participant characteristics

A total of 400 ICU nurses who met the eligibility criteria were approached and received the questionnaire. Of these, 372 questionnaires were considered valid and included in the final analysis, yielding a valid response rate of 93.0%. The remaining 28 questionnaires were excluded because of incomplete responses or failure to meet the predefined data-quality criteria. The participants ranged in age from 23 to 50 years, with a mean age of 33.19 years (SD = 5.21). Among them, 84.4% were female and 15.6% were male. The majority (73.1%) were married, and more than half had over 10 years of work experience. Detailed demographic information is presented in Table 1.

**Table 1:** Descriptions of intensive care nurse characteristics (*N* = 372).

Variables		Frequency (*n*)	Percentage (%)
Gender	Male	58	15.59
	Female	314	84.41
Marital status	Single	100	26.89
	Married	272	73.12
Education level	Junior college or below	35	9.41
	Bachelor’s	325	87.37
	Master’s or above	12	3.23
Work experience (years)	1–5	106	28.49
	6–10	64	17.20
	>10	202	54.30
Professional title	Nurse	30	8.06
	Nurse practitioner	112	30.11
	Nurse-in-charge	208	55.91
	Associate professor of nursing or above	22	5.91
Monthly income (RMB)	<5,000	70	18.82
	5,000 ~ 10,000	162	43.55
	≥10,000	140	37.63

### 4.3 Descriptive statistics and correlation analysis

Table 2 presents the descriptive statistics and correlation coefficients for the main study variables. The mean score for responsible leadership was 3.73 (SD = 0.47), and the mean score for innovative behavior was 3.16 (SD = 0.68). Perceived insider status had a mean of 3.97 (SD = 0.88), while voice behavior averaged 3.68 (SD = 0.60).

**Table 2:** Descriptive statistics and correlation analysis (*r*).

Variables	1	2	3	4	5	6	7	8	9	10	11	12	13	Mean ± SD	K-S (*p*-value)
1. RL	1													3.73 ± 0.47	0.025(0.200)
2. SDCO	0.818**	1												3.65 ± 0.60	
3. SR	0.774**	0.547**	1											3.81 ± 0.31	
4. IDM	0.790**	0.497**	0.532**	1										3.53 ± 0.50	
5. LTSO	0.788**	0.472**	0.468**	0.527**	1									3.93 ± 0.58	
6. PIS	0.277**	0.209**	0.231**	0.156**	0.280**	1								3.97 ± 0.88	0.025(0.200)
7. VB	0.291**	0.174**	0.236**	0.201**	0.316**	0.265**	1							3.68 ± 0.60	0.032(0.092)
8. PV	0.233**	0.157**	0.171**	0.171**	0.237**	0.214**	0.855**	1						3.45 ± 0.76	
9. PrV	0.255**	0.133*	0.226**	0.164**	0.295**	0.230**	0.819**	0.401**	1					3.91 ± 0.68	
10. IB	0.427**	0.333**	0.330**	0.251**	0.429**	0.438**	0.404**	0.332**	0.345**	1				3.16 ± 0.68	0.032(0.090)
11. IG	0.363**	0.270**	0.319**	0.205**	0.358**	0.276**	0.327**	0.286**	0.261**	0.722**	1			3.36 ± 0.78	
12. IP	0.289**	0.244**	0.231**	0.139**	0.291**	0.357**	0.354**	0.303**	0.289**	0.763**	0.420**	1		3.01 ± 0.66	
13. IR	0.352**	0.267**	0.237**	0.244**	0.355**	0.370**	0.288**	0.220**	0.265**	0.839**	0.415**	0.416**	1	3.11 ± 0.60	

**p < 0.01. RL, Responsible leadership; SDCO, Self-discipline and consideration for others; SR, Social responsibility; IDM, Interactive decision-making; LTSO, Long-term strategic orientation; PIS, Perceived insider status; VB, Voice behavior; PV, Prohibitive voice; PrV, Promotive voice; IB, Innovative behavior; IG, Idea generation; IP, Idea promotion; IR, Idea realization; K-S, Kolmogorov–Smirnov test. *p < 0.05, **p < 0.01.

Normality tests using the Kolmogorov–Smirnov method showed that all variables were normally distributed (*p* > 0.05), thus Pearson correlation analysis was applied. The results indicated that responsible leadership was significantly positively associated with perceived insider status, voice behavior, and innovative behavior (*r* = 0.277, 0.291, 0.427; all *p* < 0.01). Additionally, perceived insider status was positively correlated with voice behavior and innovative behavior (*r* = 0.265, 0.438; all *p* < 0.01), and voice behavior also showed a significant positive correlation with innovative behavior (*r* = 0.404, *p* < 0.01).

### 4.4 Mediating effect analysis

Structural equation modeling (SEM) was performed to test whether perceived insider status and voice behavior mediated the relationship between responsible leadership and innovative behavior. The model demonstrated an acceptable overall fit based on the goodness-of-fit indices (see Table 3).

**Table 3:** Model-fitting standard and fitting index of the final model.

Criteria	*χ^2^/df*	*GFI*	*AGFI*	*CFI*	*IFI*	*RMSEA*	*SRMR*
Model-fitting standard	<3	>0.90	>0.90	>0.90	>0.90	≤0.08	<0.05
Model-fitting index	1.74	0.96	0.94	0.97	0.97	0.05	0.03

**Figure 2:**
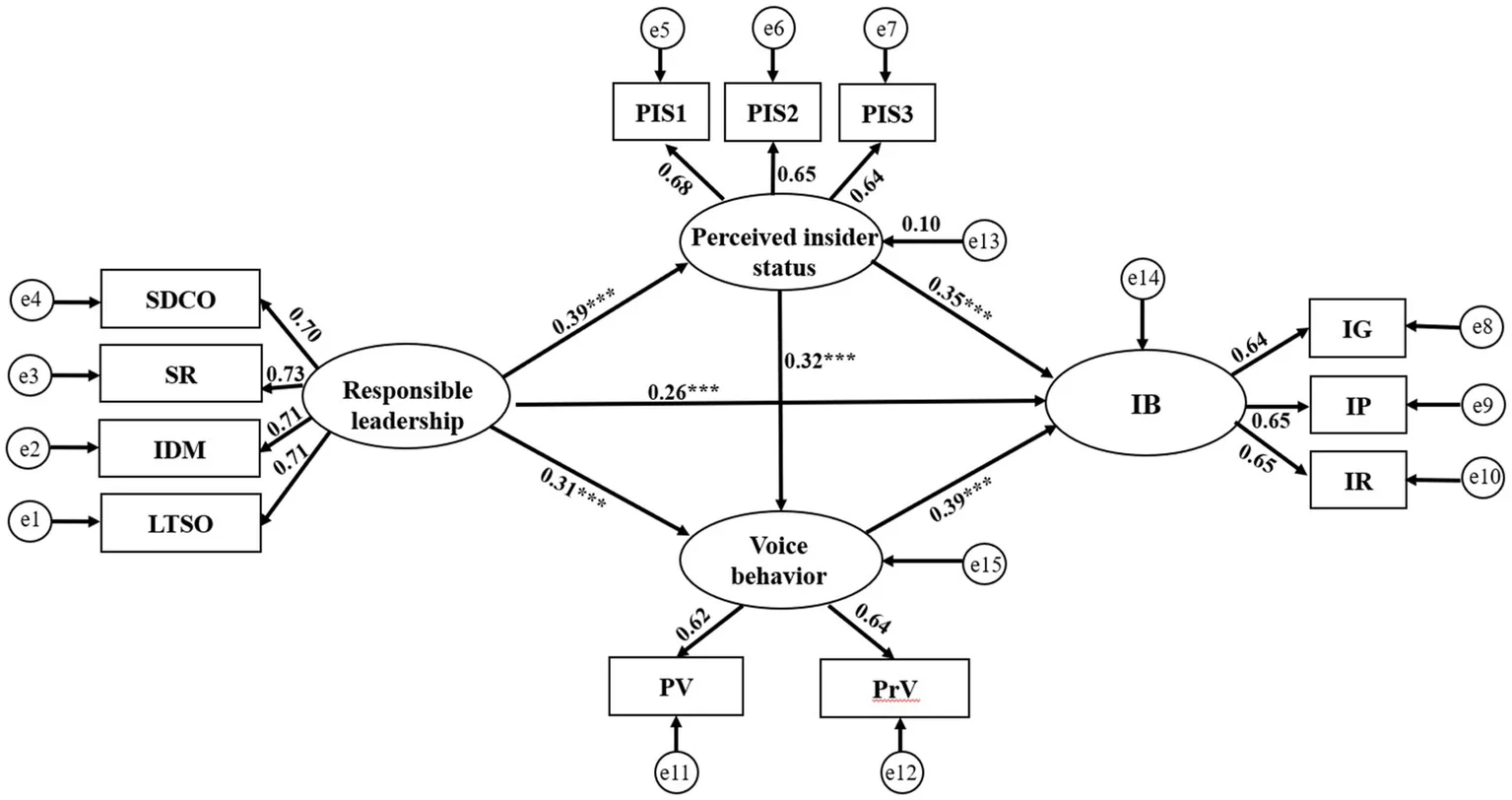
The mediating roles of perceived insider status and voice behavior between responsible and innovative behavior. SDCO, Self-discipline and consideration for others; SR, Social responsibility; IDM, Interactive decision-making; LTSO, Long-term strategic orientation; PV, Prohibitive voice; PrV, Promotive voice; IB, Innovative behavior; IG, Idea generation; IP, Idea promotion; IR, Idea realization; PIS1-3, three parcels of perceived insider status; ***p < 0.001.

The mediation analysis showed that responsible leadership was significantly and positively associated with ICU nurses’ perceived insider status (*β* = 0.39, *t* = 5.28 *p* < 0.001), voice behavior (*β* = 0.31, *t* = 3.57, *p* < 0.001), and innovative behavior (*β* = 0.26, *t* = 3.44, *p* < 0.001). Perceived insider status was positively associated with voice behavior (*β* = 0.32, *t* = 3.41, *p* < 0.001) and innovative behavior (*β* = 0.35, *t* = 4.12, *p* < 0.001). Voice behavior was also positively associated with innovative behavior (*β* = 0.39, *t* = 3.79, *p* < 0.001). These findings support the hypothesized mediating roles of perceived insider status and voice behavior in the relationship between responsible leadership and innovative behavior (see Figure 2).

To further verify the mediating effects, a bootstrap analysis with 5,000 resamples was conducted using both percentile and bias-corrected 95% confidence intervals. Following the approach recommended by Preacher and Hayes (2008), mediation was considered significant if the confidence interval did not include zero. The results indicated that perceived insider status significantly mediated the relationship between responsible leadership and innovative behavior. Additionally, voice behavior also served as a significant mediator in this relationship. Moreover, a sequential (chain) mediating effect was observed, with responsible leadership influencing innovative behavior through perceived insider status and voice behavior in turn (see Table 4).

**Table 4:** Bootstrap analysis of the mediating model.

Effect	Path	Standardized *β*	SE	The size of effect	95%CI	
					Lower	Upper
IndA1	RL → PIS → IB	0.14	0.02	22.56%	0.10	0.18
IndA2	RL → VB → IB	0.12	0.03	21.05%	0.08	0.19
IndA3	RL → PIS → VB → IB	0.05	0.02	8.77%	0.04	0.13
Direct	RL → IB	0.26				
Total	RL → IB	0.57				

RL, Responsible leadership; PIS, Perceived insider status; VB, Voice behavior; IB, Innovative behavior; SE, Standard error.

## 5 Discussion

This study investigated the association between responsible leadership and ICU nurses’ innovative behavior, emphasizing the mediating roles of perceived insider status and voice behavior. The findings indicate that responsible leadership was positively associated with nurses’ innovative behavior. Perceived insider status and voice behavior both played partial mediating roles, suggesting that nurses who feel valued as organizational members may be more likely to speak up and engage in innovative behavior. Moreover, the results supported a sequential mediation pathway linking responsible leadership to perceived insider status, voice behavior, and innovative behavior. This identity-to-action pathway extends existing research by illustrating how organizational belonging may be translated into proactive expression and innovation. These findings highlight the importance of relational and culturally responsive leadership practices in supporting frontline innovation in high-intensity clinical settings such as ICUs.

### 5.1 Effect of responsible leadership on innovative behavior

In this study, the overall score for innovative behavior among ICU nurses was higher than that reported in Wang et al.’s study on general clinical nurses in China (Wang et al., 2024). This discrepancy may stem from differences in the study populations. While Wang’s research included nurses across various departments (Wang et al., 2024), our study focused specifically on ICU nurses, who routinely operate in highly complex, time-sensitive, and technologically demanding environments. These working conditions require constant problem-solving and adaptability, which may naturally foster stronger innovation tendencies (Zhang et al., 2025).

Consistent with prior studies (Pathak and Jha, 2025), our findings confirm that responsible leadership is a significant positive predictor of ICU nurses’ innovative behavior, *supporting Hypothesis 1*. In high-pressure environments like the ICU, where patient conditions are often critical and nursing tasks are complex, leadership plays a pivotal role in shaping nurses’ motivation and behavioral outcomes (Huang H. et al., 2025). Responsible leaders emphasize ethical conduct, fairness, and mutual respect, which fosters a psychologically safe climate where nurses feel valued and empowered to express new ideas (Lin et al., 2020). This inclusive and trust-based environment encourages nurses to challenge existing practices and actively seek improvements in care delivery. Furthermore, by balancing organizational goals with the needs of frontline staff, responsible leadership promotes open dialog, problem-solving, and collective responsibility—all of which contribute to enhancing innovation at the unit level (Han and Ni, 2025). These findings suggest that cultivating responsible leadership in intensive care settings may be a key strategy to unlocking the innovation potential of clinical nursing teams.

### 5.2 Mediation through perceived insider status

The study found that participants’ perceived insider status is consistent with findings from prior research (Stamper and Masterson, 2002) indicating that ICU nurses generally recognize themselves as valued and integral members of their organizations. This sense of belonging plays a crucial role in fostering positive work attitudes and behaviors. The present findings further suggest that perceived insider status plays a significant mediating role in the relationship between responsible leadership and ICU nurses’ innovative behavior, *supporting Hypothesis 2*. When nurses perceive themselves as valued members of the organization, they are more likely to engage in behaviors that contribute to organizational improvement (Wang and Kim, 2013). In the high-stakes context of intensive care, where collaboration, adaptability, and trust are essential, feeling like an “insider” enhances nurses’ sense of ownership and psychological security, which are key drivers of innovation (Zhang G. et al., 2024). Responsible leadership fosters such perceptions by promoting fairness, mutual respect, and inclusive decision-making (Wang and Kim, 2013). This leadership style affirms the contributions of frontline nurses, making them feel respected, trusted, and empowered to take initiative (Mohebi et al., 2022). In Chinese cultural settings, where relational harmony and collective identity are emphasized, perceived insider status is particularly important in motivating staff to go beyond routine tasks (Wang and Kim, 2013). Nurses who identify strongly with their organization are more inclined to generate and implement new ideas, contributing to continuous improvement in care quality. Thus, enhancing insider identity may be a critical pathway through which responsible leadership stimulates innovative behavior in clinical practice.

### 5.3 Mediation through voice behavior

In this study, participants’ voice behavior was found to be at a moderate level, consistent with previous findings in similar clinical contexts (Lu et al., 2025). This suggests that ICU nurses are generally willing to express their opinions and suggestions in the workplace. However, there is still room for improvement. This may be influenced by hierarchical organizational structures and the high-risk nature of ICU settings, which can sometimes inhibit open communication (Nembhard and Edmondson, 2006). The results further confirm that responsible leadership not only directly promotes innovative behavior, but also exerts an indirect effect through the partial mediating role of voice behavior, *supporting Hypothesis 3*. Specifically, responsible leaders emphasize fairness, trust-building, and inclusive dialog, which helps create a psychologically safe environment where nurses feel encouraged to speak up. In such an atmosphere, nurses are more likely to express constructive ideas aimed at improving clinical practices. Moreover, responsible leadership reinforces team collaboration and empowers nurses to participate actively in decision-making, thereby enhancing their engagement and voice (Uzum et al., 2025). When nurses are given both the opportunity and confidence to share their perspectives, their proactive contributions can significantly stimulate innovation in ICU care.

### 5.4 Mediation through perceived insider status and voice behavior

This study also revealed that perceived insider status significantly influences ICU nurses’ voice behavior, which aligns with existing research in organizational behavior (Chen, 2021). Furthermore, we found that in the relationship between responsible leadership and innovative behavior, perceived insider status and voice behavior jointly serve as sequential mediators, *supporting Hypothesis 4*.

ICU nurses often work in high-pressure, high-risk environments where clear communication and proactive engagement are critical. Previous studies have shown that when nurses perceive themselves as insiders—valued and trusted members of the organization—they are more likely to speak up with constructive suggestions that enhance team functioning and patient safety (Gong et al., 2021). Responsible leadership plays a vital role in fostering this perception by emphasizing fairness, mutual respect, and participatory management (Voegtlin et al., 2012). Once nurses feel psychologically included, their willingness to express new ideas and voice concerns increases significantly (Mohebi et al., 2022). This enhanced voice behavior not only contributes to process improvements but also becomes a channel through which insider identity is transformed into practical innovation. Therefore, under responsible leadership, a sense of insider status can stimulate nurses to engage more actively in voice behavior, which ultimately facilitates innovative practices in intensive care settings.

## 6 Limitations and future research

This study provides insights into the relationships among responsible leadership, perceived insider status, voice behavior, and innovative behavior among ICU nurses. However, several limitations should be acknowledged. First, participants were recruited through convenience sampling from only three tertiary hospitals in northern China, which limits the representativeness and generalizability of the findings. Differences in organizational culture, leadership structures, nurse staffing patterns, workload, and healthcare resources across regions and hospital levels may shape nurses’ perceptions of leadership, organizational belonging, and willingness to voice or implement new ideas. Therefore, the present findings should not be generalized to ICU nurses nationwide without caution. Future studies should adopt multi-center, stratified sampling designs involving hospitals of different levels across northern, central, southern, and western China.

Second, the sample was predominantly female, with male nurses accounting for only 15.6% of participants. This imbalance limits our ability to determine whether the proposed relationships operate similarly across genders and may reduce the representativeness of the findings for male ICU nurses. Given the relatively small male subgroup, a subgroup mediation analysis may yield unstable estimates and insufficient statistical power. Future studies should recruit larger and more gender-balanced samples, using gender-stratified sampling where feasible, to examine potential gender differences in the proposed mediation pathways.

Third, the cross-sectional design limits the ability to establish temporal ordering or infer causal relationships among the variables. Longitudinal or experimental studies are needed to examine changes over time and further validate the proposed mediation pathways. Finally, alternative or reciprocal relationships among responsible leadership, perceived insider status, voice behavior, and innovative behavior cannot be excluded. Future research could compare competing models and incorporate longitudinal or mixed-method designs to provide a more comprehensive understanding of these relationships.

## 7 Implications for clinical practice

The findings suggest that practical strategies may be implemented at both the hospital administrative and ICU ward levels to support the proposed pathways linking responsible leadership, perceived insider status, voice behavior, and innovative behavior. At the hospital administrative level, nursing departments should establish structured responsible leadership development programs for nurse managers, including scenario-based training, leadership coaching, and regular feedback. Training should focus on ethical decision-making, stakeholder engagement, inclusive communication, and constructive responses to staff concerns. Hospitals may also establish formal mechanisms to strengthen nurses’ organizational belonging, such as regular staff consultation meetings, nurse participation in clinical decision-making, and recognition or reward programs for nursing innovation. In addition, protected channels for reporting concerns and proposing improvements, together with clear non-retaliation policies and timely managerial feedback, may help create an organizational environment in which nurses feel safer expressing constructive ideas.

At the ICU ward level, nurse managers should translate these organizational strategies into routine practice. Regular staff opinion meetings, brief safety huddles, leadership rounds, and participation in ward-level quality-improvement activities may reinforce nurses’ sense of being valued organizational members. To facilitate voice behavior in a hierarchical clinical environment, ICU supervisors should actively invite suggestions, provide anonymous channels when necessary, use non-punitive responses to concerns and errors, and provide timely feedback on how nurses’ suggestions are addressed. Feasible ideas can be supported through small-scale pilot projects, with contributors acknowledged for their involvement. Together, these practices may strengthen perceived insider status, facilitate constructive voice, and support the translation of nurses’ ideas into innovative clinical practices.

## 8 Conclusion

In the context of rising healthcare demands and nursing shortages in China, enhancing the innovative behavior of ICU nurses has become increasingly vital for sustaining high-quality critical care. This study demonstrated that responsible leadership not only directly promotes ICU nurses’ innovative behavior, but also indirectly influences it through the mediating roles of perceived insider status and voice behavior. These findings offer important insights into the psychological mechanisms by which leadership impacts innovation, particularly within the hierarchical and collectivist cultural context of Chinese hospitals. By fostering responsible leadership and building inclusive organizational climates, nursing managers can more effectively mobilize the creativity and initiative of ICU nurses, thereby contributing to improved care quality and healthcare system resilience.

The de-identified data supporting the findings of this study will be available from the corresponding author upon reasonable request following publication, subject to ethical and institutional requirements. Identifiable or sensitive personal, clinical, and institutional information will not be shared.

## Data Availability

The raw data supporting the conclusions of this article will be made available by the authors, without undue reservation.

## References

[ref1] Alluhaybi A. Usher K. Durkin J. Wilson A. (2024). Clinical nurse managers' leadership styles and staff nurses' work engagement in Saudi Arabia: a cross-sectional study. PLoS One 19:e0296082. doi: 10.1371/journal.pone.0296082, 38452098 PMC10919612

[ref2] Byrne B. M. (2010). Structural Equation Modeling with AMOS: Basic Concepts, Applications, and Programming. 2nd Edn New York, NY, US: Routledge/Taylor & Francis Group.

[ref3] Chen L. (2021). Differential leadership, organizational identity, and employee voice behavior--the moderating role of perceived insider status. Sci. J. Econ. Manage. Res. Volume 3.

[ref4] Chen A. S. Y. Hou Y. H. (2016). The effects of ethical leadership, voice behavior and climates for innovation on creativity: a moderated mediation examination. Leadersh. Q. 27, 1–13. doi: 10.1016/j.leaqua.2015.10.007

[ref5] Cheng X. Chen H. Zheng M. (2021). The structure dimension and scale development of responsible leadership in the Chinese cultural context. Chin. J. Manag. 18, 1780–1789.

[ref6] Elbus L. M. S. Mostafa M. G. Mahmoud F. Z. Shaban M. Mahmoud S. A. (2024). Nurse managers' managerial innovation and it's relation to proactivity behavior and locus of control among intensive care nurses. BMC Nurs. 23:485. doi: 10.1186/s12912-024-02084-8, 39014395 PMC11251221

[ref7] Gong Z. Van Swol L. M. Li F. Gilal F. G. (2021). Relationship between nurse's voice and self-leadership: a time-lagged study. Nurs. Open 8, 1038–1047. doi: 10.1002/nop2.711 PMC8046052 33393229

[ref8] Guo J. Qiu Y. Gan Y. (2022). Workplace incivility and work engagement: the chain mediating effects of perceived insider status, affective organizational commitment and organizational identification. Curr. Psychol. 41, 1809–1820. doi: 10.1007/s12144-020-00699-z

[ref9] Guzman F. A. Espejo A. (2019). Introducing changes at work: how voice behavior relates to management innovation. J. Organ. Behav. 40, 73–90. doi: 10.1002/JOB.2319

[ref10] Haider S. A. Akbar A. Tehseen S. Poulova P. Jaleel F. (2022). The impact of responsible leadership on knowledge sharing behavior through the mediating role of person–organization fit and moderating role of higher educational institute culture. J. Innov. Knowl. 7:100265. doi: 10.1016/j.jik.2022.100265

[ref11] Han Z. Ni M. (2025). Effects of responsible leadership on employee innovative behavior: the role of knowledge sharing and psychosocial safety climate. Leadersh. Organ. Dev. J. 46, 524–539. doi: 10.1108/LODJ-09-2023-0473

[ref12] Han Z. Wang Q. Yan X. (2019a). How responsible leadership motivates employees to engage in organizational citizenship behavior for the environment: a double-mediation model. Sustainability 11:605. doi: 10.3390/su11030605

[ref13] Han Z. Wang Q. Yan X. (2019b). How responsible leadership predicts organizational citizenship behavior for the environment in China. Leadersh. Organ. Dev. J. 40, 305–318. doi: 10.1108/LODJ-07-2018-0256

[ref14] Han Z. Zhu Z. Wang D. Wei W. (2024). Responsible leadership and employee ethical voice: mediating role of ethical efficacy and moderating role of moral identity. Curr. Psychol. 43, 29516–29527. doi: 10.1007/s12144-024-06564-7

[ref15] Huang S. Chen H. Zhang L. Weng X. Zhou L. Ma X. . (2025). Investigation of potential profiles and influencing factors of voice behavior among Chinese nurses. BMC Nurs. 24:150. doi: 10.1186/s12912-025-02786-7, 39923084 PMC11807296

[ref16] Huang H. Zhang X. Tu L. Peng W. Wang D. Chong H. . (2025). Inclusive leadership, self-efficacy, organization-based self-esteem, and intensive care nurses' job performance: a cross-sectional study using structural equation modeling. Intensive Crit. Care Nurs. 87:103880. doi: 10.1016/j.iccn.2024.103880, 39500700

[ref17] Hui C. Lee C. Wang H. (2015). Organizational inducements and employee citizenship behavior: the mediating role of perceived insider status and the moderating role of collectivism. Hum. Resour. Manag. 54, 439–456. doi: 10.1002/hrm.21620

[ref18] Iacobucci D. (2010). Structural equations modeling: fit indices, sample size, and advanced topics. J. Consum. Psychol. 20, 90–98. doi: 10.1016/j.jcps.2009.09.003

[ref19] Identity R. (2007). Understanding responsible leadership: role identity and motivational drivers Nicola M. Pless. J. Bus. Ethics 74, 437–456. doi: 10.2307/25075481

[ref20] Kline R. B. (2023). Principles and Practice of Structural Equation Modeling. New York: Guilford Publications.

[ref21] Landis R. S. Beal D. J. Tesluk P. E. (2000). A comparison of approaches to forming composite measures in structural equation models. Organ. Res. Methods 3, 186–207. doi: 10.1177/109442810032003

[ref22] Lee J. Oh S.-H. Park S. (2022). Effects of organizational embeddedness on unethical pro-organizational behavior: roles of perceived status and ethical leadership. J. Bus. Ethics 176, 111–125. doi: 10.1007/s10551-020-04661-8

[ref23] Li Y. Sun J.-M. (2015). Traditional Chinese leadership and employee voice behavior: a cross-level examination. Leadersh. Q. 26, 172–189. doi: 10.1016/j.leaqua.2014.08.001

[ref24] Liang J. Farh C. I. Farh J.-L. (2012). Psychological antecedents of promotive and prohibitive voice: a two-wave examination. Acad. Manag. J. 55, 71–92. doi: 10.5465/amj.2010.0176

[ref25] Lin C.-P. Huang H.-T. Huang T. Y. (2020). The effects of responsible leadership and knowledge sharing on job performance among knowledge workers. Pers. Rev. 49, 1879–1896. doi: 10.1108/PR-12-2018-0527

[ref26] Ling B. Lin W. Ya-qing Z. (2012). Development and analysis of reliability and validity of nurse innovative behavior scale. J. Shanghai Jiaotong Univ. (Med. Sci.) 32:1079.

[ref27] Liu B. Zhao H. Wang Y. Lu Q. (2021). The influence of team mindfulness on nurses' presenteeism: a cross-sectional study from the perspective of sensemaking. J. Nurs. Manag. 29, 1668–1678. doi: 10.1111/jonm.13289, 33605474

[ref28] Li-Ying J. Paunova M. Egerod I. (2016). Knowledge sharing behaviour and intensive care nurse innovation: the moderating role of control of care quality. J. Nurs. Manag. 24, 943–953. doi: 10.1111/jonm.12404 27271179

[ref29] Lu M. Xia R. Wang R. Zou X. (2025). Network analysis of the relationships between voice behavior and team psychological safety climate among Chinese nurses. BMC Nurs. 24:794. doi: 10.1186/s12912-025-03388-z, 40598063 PMC12211131

[ref30] Ma Z. Huang Y. Wu J. Dong W. Qi L. (2014). What matters for knowledge sharing in collectivistic cultures? Empirical evidence from China. J. Knowl. Manag. 18, 1004–1019. doi: 10.1108/JKM-06-2014-0252

[ref31] Maak T. Pless N. M. (2006). Responsible leadership in a stakeholder society–a relational perspective. J. Bus. Ethics 66, 99–115. doi: 10.1007/s10551-006-9047-z

[ref32] Miska C. Mendenhall M. E. (2018). Responsible leadership: a mapping of extant research and future directions. J. Bus. Ethics 148, 117–134. doi: 10.1007/s10551-015-2999-0

[ref33] Mohebi M. M. Zandi K. Fotovat H. K. (2022). Relationship between responsible leadership and caring behaviors of nurses: the mediating role of organizational pride. Avicenna J. Nurs. Midwifery Care 30, 297–306. doi: 10.32592/ajnmc.30.4.297

[ref34] Mousa M. Arslan A. (2023). Responsible leadership practices in the hospitality sector family businesses: evidence from an emerging market. J. Fam. Bus. Manage. 13, 1429–1442. doi: 10.1108/JFBM-01-2023-0008

[ref35] Nembhard I. M. Edmondson A. C. (2006). Making it safe: the effects of leader inclusiveness and professional status on psychological safety and improvement efforts in health care teams. J. Organ. Behav. 27, 941–966. doi: 10.1002/job.413

[ref36] Ni P. Chen J. Liu N. (2010). The sample size estimation in quantitative nursing research. Chin. J. Nurs. 45, 378–380.

[ref37] Pathak P. Jha S. (2025). Responsible leadership behavioral styles and innovative work behavior: a moderated-mediation analysis. Glob. Bus. Organ. Excell. 44, 44–58. doi: 10.1002/joe.22282

[ref38] Podsakoff P. M. MacKenzie S. B. Lee J.-Y. Podsakoff N. P. (2003). Common method biases in behavioral research: a critical review of the literature and recommended remedies. J. Appl. Psychol. 88, 879–903. doi: 10.1037/0021-9010.88.5.879, 14516251

[ref39] Preacher K. J. Hayes A. F. (2008). Asymptotic and resampling strategies for assessing and comparing indirect effects in multiple mediator models. Behav. Res. Methods 40, 879–891. doi: 10.3758/brm.40.3.879, 18697684

[ref40] Qi L. Liu B. (2017). Effects of inclusive leadership on employee voice behavior and team performance: the mediating role of caring ethical climate. Front. Commun. 2:8. doi: 10.3389/fcomm.2017.00008

[ref41] Saleh M. S. M. Ata A. A. Abd-Elhamid Z. N. Eltahan A. A. Dailah H. G. Elsabahy H. E. (2025). Building nursing leaders: the influence of entrepreneurial leadership program on nurse interns' innovation and clinical performance. BMC Nurs. 24:501. doi: 10.1186/s12912-025-03100-1, 40340834 PMC12063326

[ref42] Scully N. J. (2015). Leadership in nursing: the importance of recognising inherent values and attributes to secure a positive future for the profession. Collegian 22, 439–444. doi: 10.1016/j.colegn.2014.09.004, 26775531

[ref43] Shalendra S. Jeon D. Lingam S. Pritam A. Ku B. (2021). Can insider status promote employee voice behavior? Int. J. Manage. Sustaina 10, 123–134. doi: 10.18488/journal.11.2021.104.123.134

[ref44] Stamper C. L. Masterson S. S. (2002). Insider or outsider? How employee perceptions of insider status affect their work behavior. J. Organ. Behav. 23, 875–894. doi: 10.1002/job.175

[ref45] Stewart M. Fortin M. Brown J. B. Ryan B. L. Pariser P. Charles J. . (2021). Patient-centred innovation for multimorbidity care: a mixed-methods, randomised trial and qualitative study of the patients' experience. Br. J. Gen. Pract. 71, e320–e330. doi: 10.3399/bjgp21X714293, 33753349 PMC7997674

[ref46] Tuan L. T. (2022). Promoting employee green behavior in the Chinese and Vietnamese hospitality contexts: the roles of green human resource management practices and responsible leadership. Int. J. Hosp. Manag. 105:103253. doi: 10.1016/j.ijhm.2022.103253

[ref47] Uzum B. Ozkan O. S. Ozkurt Sivrikaya S. Ciftyildiz K. (2025). Does responsible leadership via voice behavior promote green behavior? Evid. Based HRM Glob. Forum Empir. Scholarsh. 14, 223–238. doi: 10.1108/EBHRM-04-2024-0133

[ref48] Voegtlin C. Patzer M. Scherer A. G. (2012). Responsible leadership in global business: a new approach to leadership and its multi-level outcomes. J. Bus. Ethics 105, 1–16. doi: 10.1007/s10551-011-0952-4

[ref49] Wang J. Kim T. Y. (2013). Proactive socialization behavior in China: the mediating role of perceived insider status and the moderating role of supervisors' traditionality. J. Organ. Behav. 34, 389–406. doi: 10.1002/job.1811

[ref50] Wang Z. Yang L. Zhu Y. Tang X. Wang T. Chen L. . (2024). Innovative behavior and structural empowerment among the Chinese clinical nurses: the mediating role of decent work perception. BMC Nurs. 23:881. doi: 10.1186/s12912-024-02554-z, 39627823 PMC11613594

[ref51] Wijewardena N. Samaratunge R. Härtel C. (2014). Creating better employees through positive leadership behavior in the public sector. Int. J. Public Adm. 37, 288–298. doi: 10.1080/01900692.2013.835320

[ref52] Yan D. Wen F. Li X. Zhang Y. (2020). The relationship between psychological capital and innovation behaviour in Chinese nurses. J. Nurs. Manag. 28, 471–479. doi: 10.1111/jonm.12926, 31811781

[ref53] Yan A. Xiao Y. (2016). Servant leadership and employee voice behavior: a cross-level investigation in China. Springerplus 5:1595. doi: 10.1186/s40064-016-3264-4, 27652168 PMC5026985

[ref54] Yuan S. Jiang R. (2024). Perceived insider status and innovative behaviour of the new generation of employees: mediating effect of innovative self-efficacy and moderation of group competitive climate. J. Psychol. Afr. 34, 52–58. doi: 10.1080/14330237.2023.2290431

[ref55] Yue Y. Lin W. L. Tan H. C. (2025). Unlocking employee creativity: ethical leadership, employee voice behavior and innovation climate in the era of intelligent manufacturing in China. Soc. Responsib. J. 21, 1440–1465. doi: 10.1108/SRJ-11-2024-0813

[ref56] Zhang X. Huang H. Zhao S. Li D. Du H. (2025). Emotional exhaustion and turnover intentions among young ICU nurses: a model based on the job demands-resources theory. BMC Nurs. 24:136. doi: 10.1186/s12912-025-02765-y, 39915810 PMC11800589

[ref57] Zhang S. Liu Y. Li G. Zhang Z. Fa T. (2022). Chinese nurses' innovation capacity: the influence of inclusive leadership, empowering leadership and psychological empowerment. J. Nurs. Manag. 30, 1990–1999. doi: 10.1111/jonm.13654, 35476276

[ref58] Zhang K. Wang Y. Tang N. (2024). Power distance orientation and perceived insider status in China: a social identity perspective. Asia Pac. Bus. Rev. 29, 89–113. doi: 10.1080/13602381.2022.2082115, 37339054

[ref59] Zhang G. Zhang X. Wang Y. (2024). Perceived insider status and employees' innovative behavior: the role of knowledge sharing and organizational innovation climate. Eur. J. Innov. Manag. 27, 589–607. doi: 10.1108/EJIM-03-2022-0123

[ref60] Zhao J. Wu X. Chen Y. Li T. Han Y. Liu T. . (2023). What makes a hospital excellent? A qualitative study on the organization and management of five leading public hospitals in China. Risk Manage. Healthc. Policy 16, 1915–1927. doi: 10.2147/RMHP.S424711, 37746043 PMC10516193

[ref61] Zhong X. Fu Y. Wang T. (2019). Inclusive leadership, perceived insider status and employee knowledge sharing——moderating role of organizational innovation climate. RD Manage 31, 109–120.

